# Efficacy of platinum-based adjuvant chemotherapy in T2aN0 stage IB non-small cell lung cancer

**DOI:** 10.1186/1749-8090-8-151

**Published:** 2013-06-11

**Authors:** Seong Yong Park, Jin Gu Lee, Jieun Kim, Go Eun Byun, Mi Kyung Bae, Chang Young Lee, Dae Joon Kim, Kyung Young Chung

**Affiliations:** 1 Department of Thoracic and Cardiovascular Surgery, Yonsei University College of Medicine, 50 Yonsei-ro, Seodaemun-gu, Seoul 120-752, Republic of Korea; 2 Biostatistics Collaboration Unit, Yonsei University College of Medicine, Seoul, Republic of Korea

**Keywords:** Lung cancer surgery, Adjuvant therapy, Statistics, Survival analysis

## Abstract

**Background:**

Although overall survival for non-small cell lung cancer (NSCLC) has increased, survival rate for pathologically staged T2aN0M0 stage IB NSCLC remains low. Adjuvant chemotherapy is not a standard treatment for stage IB NSCLC. Our purpose was to determine the efficacy of platinum-based adjuvant chemotherapy in stage IB NSCLC.

**Methods:**

We retrospectively reviewed the medical records of 119 stage IB patients who underwent lobectomy and mediastinal lymph node dissection. Among these, 60 patients underwent platinum-based adjuvant chemotherapy (adjuvant group) and 59 did not receive chemotherapy (observation group).

**Results:**

Participants had a mean age of 62.12 ± 11.51 years and 73 (61.3%) were male. The median follow-up period was 49.04 months. Mean age was higher in the observation group whereas patients in the adjuvant group had larger tumors, more dissected lymph nodes, and better performance status. The 5-year overall survival was 64.7% in the observation group and 88.2% in the adjuvant group (*p* = 0.010). The 5-year disease-free survival was 51.3% in the observation group and 74.0% in the adjuvant group (*p* = 0.011). In multivariate analysis, only platinum-based adjuvant chemotherapy was a risk factor for overall survival [hazard ratio (HR) = 0.428, *p* = 0.049] and disease-free survival (HR = 0.57, *p* = 0.043). In subset analysis, patients with a larger tumor (greater than 3.2 cm), moderate to poor differentiation, and good performance status (Eastern Cooperative Oncology Group, 0) benefitted from platinum-based adjuvant chemotherapy.

**Conclusions:**

Platinum-based adjuvant chemotherapy for surgically treated stage IB NSCLC might offer better survival than observation alone. A large-scale randomized clinical trial is needed to validate these findings.

## Background

Lung cancer is the leading cause of cancer deaths among men and women worldwide [1]. Fewer than 20% of patients diagnosed with lung cancer are candidates for surgical resection. Among patients who are candidates for surgical resection, the proportion with early stage non-small cell lung cancer (NSCLC) has increased due to surveillance programs and use of low-dose computed tomography for screening [2]. The mainstay of treatment for stage I NSCLC is complete surgical resection with mediastinal lymph node dissection. However, although the overall survival for stage IA NSCLC has been reported to be up to 73-90% with surgical treatment only [3,4], the overall survival for pathologically staged T2aN0M0 stage IB NSCLC remains low, at approximately 60% [4]. This modest and unsatisfactory result for node-negative NSCLC has been a source of frustration, and the possible use of adjuvant treatment after surgical resection for stage IB NSCLC has recently attracted interest among thoracic surgeons and oncologists.

Many randomized clinical trials have reported the efficacy of platinum-based adjuvant chemotherapy after surgical resection in stage II–IIIA lung cancer [5-7]. The efficacy of platinum-based adjuvant chemotherapy in stage IB NSCLC also has been studied in a clinical trial [8] but the final results did not demonstrate clear benefits of adjuvant chemotherapy. Adjuvant chemotherapy is not currently a standard treatment for stage IB NSCLC and its role in stage IB disease remains controversial. Recent National Comprehensive Cancer Network (NCCN) guidelines recommend adjuvant chemotherapy with low-level evidence (category 2B) in patients with stage IB NSCLC and risk factors for poor survival. In our institution we have performed adjuvant chemotherapy if patients with risk factors such as poor differentiation, lymphovascular invasion and pleural invasion, and patients were considered able to tolerate platinum-based adjuvant chemotherapy under the patient’s agreement on the adjuvant therapy. Therefore, our experience provides an opportunity to retrospectively evaluate the efficacy of platinum-based adjuvant chemotherapy in stage IB NSCLC patients who underwent complete surgical resection and systemic mediastinal lymph node dissection.

## Methods

### Patients

This retrospective study was approved by the institutional review board of the hospital (IRB No. 4-2012-0931). We enrolled 119 patients who were confirmed to have pathologic T2aN0 stage IB NSCLC after lobectomy and complete lymph node dissection between January 2000 and December 2009. All patients underwent R0 resection without neoadjuvant therapy and none received adjuvant radiotherapy. We retrospectively reviewed the medical records and pathologic data of these patients.

Chest computed tomography (CT), whole-body bone scan, brain magnetic resonance imaging, bronchoscopy, and pulmonary function testing were performed preoperatively in all patients. Positron emission tomography (PET) was performed from approximately 2005 onwards. Postoperatively, we examined patients at 3-month intervals for the first 2 years and at 6-month intervals thereafter on an outpatient basis. The follow-up continued for at least 5 years. The follow-up evaluation included physical examination, chest radiography, and blood tests. Chest CT was obtained at 6-month intervals, and positron emission tomography scans were obtained annually to detect recurrences. If patients had any symptoms or signs of recurrence additional imaging studies were performed regardless of the follow-up schedule. Comorbidities were defined when patients had preoperatively diagnosed cardiovascular disease (hypertension, coronary artery obstructive disease and arrhythmia), cerebrovascular disease, pulmonary disease (tuberculosis, chronic obstructive pulmonary disease and asthma) and diabetes mellitus. Performance status was recorded according to the Eastern Cooperative Oncology Group (ECOG) performance scale [9]. Local recurrences were defined as those occurring on resection margins, such as bronchial stumps or stapler lines. Regional recurrences were defined as those occurring in the hilar or mediastinal lymph nodes, pleural cavity, and ipsilateral lung. Distant recurrences were defined as those occurring in the contralateral lung, brain, liver, adrenal glands, bone, and other locations.

### Histopathologic reviews

Pathologic stages were diagnosed based on the Tumor, Node, Metastasis (TNM) Classification of the International Union Against Cancer, seventh edition [4]. All patients were reclassified based on Seventh edition of the TNM classification was published in 2007. The pathologic features recorded included the following: tumor cell type, degree of differentiation, tumor size by gross surgical specimen, lymphovascular invasion, and pleural invasion. When cancer cells were observed in the intratumoral lumen (i.e., vessels or lymphatics), the specimen was considered positive for lymphovascular invasion. Because precise differentiation between vascular and lymphatic invasion is difficult, we combined both into lymphovascular invasion. When tumors abutted the visceral pleura or when pleura were puckered, elastic-van-Gieson staining was performed. Pleural invasion was classified according to the following previously described criteria [10]: pl0, tumor with no pleural involvement beyond its elastic layer; pl1, tumor that extended beyond the elastic layer of the visceral pleura but was not exposed on the pleural surface; pl2, tumor that was exposed on the pleural surface but did not involve the adjacent anatomic structures; and pl3, tumor that involved adjacent anatomic structures.

### Adjuvant chemotherapy

The adjuvant chemotherapy was performed if patients with risk factors such as poor differentiation, lymphovascular invasion and pleural invasion, and patients were considered able to tolerate platinum-based adjuvant chemotherapy under the patient’s agreement. Platinum-based adjuvant chemotherapy was started within 4 to 8 weeks after surgical resection. Among the 60 patients who received adjuvant chemotherapy, four cycles of cisplatin (80 mg/m^2^) with paclitaxel or vinorelbine chemotherapy were performed in 30 (50%) patients and four cycles of carboplatin (an area under the curve dose of 6 mg/mL per minute over 60 minutes) with paclitaxel or vinorelbine were performed in the remaining 30 (50%). Among the 60 patients who underwent adjuvant chemotherapy, there was no mortality related to chemotherapy. Seven (11.7%) patients suffered from complications related to chemotherapy (pneumonia 3, nausea and vomiting 4) and they could not complete the planned chemotherapy. Fifty three (88.3%) completed the planned chemotherapy Decisions about dose reduction or dose delay were made by the treating medical oncologist at the time of the scheduled dose using objective criteria (white cell count, absolute neutrophil count, serum creatinine, gastrointestinal symptoms, and neurologic symptoms) and subjective criteria (performance status) [11].

### Statistical analysis

Statistical analysis was performed using STATA 11 software (Stata Corp., Stata Statistical Software, Release 11 (2005, College Station. TX). Groups were compared using the chi-square test or Fisher’s exact test for categorical variables and t-test for continuous variables. Overall survival was calculated from the date of operation to the date of death from any cause or the last follow-up. Disease-free survival was calculated from the date of operation to the date of the first recurrence or last follow-up. Other second primary malignancies were not included as an event of disease-free survival. The Kaplan-Meier method and log-rank test were used to perform univariate survival analysis and the Cox proportional hazard model was used to identify independent prognostic factors. The parameters were included in the Cox proportional hazard model if the *p*-value was less than 0.2 on the log-rank test. The criterion for significance was *p* less than 0.05 and all *p* values were derived from two-tailed tests. All statistical analysis was reviewed and verified by a statistician.

## Results

### General characteristics and pathologic results

Among the 119 patients, there were 73 male (61.3%) and 46 female patients (38.7%) with a mean age of 62.12 ± 11.51 years. The median follow-up period was 49.04 months (range, 0.3-144.84 months). The mean and median tumor size were 3.11 ± 1.01 cm and 3.2 cm (range, 0.8-4.8 cm), respectively. Sixty patients (50.4%) underwent platinum-based adjuvant chemotherapy (adjuvant group) and 59 (49.6%) patients did not receive adjuvant therapy (observation group). The general characteristics and comparisons of the two groups are shown in Table 1. Mean age was higher in the observation group than the adjuvant group (64.14 ± 12.75 vs. 59.80 ± 9.70, *p* = 0.027), whereas tumor size was smaller (28.35 ± 9.74 vs. 33.80 ± 9.82, *p* = 0.003) and the numbers of dissected lymph nodes was lower (22.88 ± 9.21 vs. 28.08 ± 13.08, *p* = 0.017). Performance status was better in the adjuvant group.

**Table 1:** General characteristics of patients

	**No adjuvant therapy (n = 59)**	**Platinum-based chemotherapy (n = 60)**	***p *value**
Age (years)	64.14 ± 12.75	59.80 ± 9.70	0.027
Male	36 (61.0%)	37 (61.7%)	0.546
FEV1 (liters)	90.10 ± 25.99	97.91 ± 25.40	0.1
Comorbidity	38 (64.4%)	33 (55.0%)	0.352
Type of operation			0.489
Lobectomy	54 (91.5%)	56 (93.3%)	
Pneumonectomy	5 (8.5%)	4 (6.7%)	
Dissected lymph nodes	22.88 ± 9.21	28.08 ± 13.08	0.017
Tumor size (mm)	28.35 ± 9.74	33.80 ± 9.82	0.003
Pathology			0.295
Adenocarcinoma	27 (45.8%)	36 (60.0%)	
Squamous cell carcinoma	18 (30.5%)	14 (23.3%)	
Others	14 (23.7%)	10 (16.7%)	
Differentiation			0.306
Well	17 (28.8%)	12 (20.0%)	
Moderate to poor	37 (62.7%)	39 (65.0%)	
Not defined	5 (8.5%)	9 (15.0%)	
Lymphovascular invasion	8 (13.6%)	11 (18.3%)	0.323
Pleural invasion			0.428
pl0	34 (57.6%)	28 (46.7%)	
pl1	15 (25.4%)	17 (28.3%)	
pl2	10 (16.9%)	15 (25.0%)	
Performance status (ECOG)			0.004
0	36 (61.0%)	52 (86.7%)	
1	20 (33.9%)	8 (13.3%)	
2	3 (5.1%)	0	

### Patterns of recurrence and death during the follow-up period

Recurrence was reported in 29 (24.4%) patients as follows: local recurrence (n = 2, 6.9%; both bronchial stump); regional recurrence (n = 11, 37.9%; 2 pleural mass, 2 mediastinal lymph node, and 7 ipsilateral lung); and distant recurrences (n = 16, 55.2%; 6 contralateral lung, 5 brain, 1 liver, 1 abdominal lymph node, and 3 bone). In adjuvant group, recurrences were reported in 12 (20%) patients; no local recurrence, 6 regional recurrences and 6 distant metastases. In observation group, 17 (28.8%) patients suffered from recurrences; 2 local recurrences, 5 regional recurrences and 6 distant metastases. The patterns of recurrence according to adjuvant chemotherapy were not different (Chi-square test, p = 0.317).

Thirty patients died during the follow-up period. Half of patients (n = 15) were died of cancer-related death after recurrence and half of patients were died of cancer non-related death. Among the most frequent cause of death was second primary cancer and pneumonia during follow-up periods (3 patients respectively).

### Overall survival and recurrence-free survival according to platinum-based adjuvant chemotherapy

**Figure 1:**
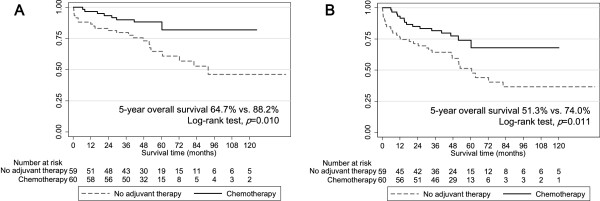
**Survival curves. A**. Overall survival curves according to adjuvant therapy. **B**. Disease-free survival curves according to adjuvant therapy.

According to the Kaplan survival model and log-rank test, the 5-year overall survival was 75.6% for all patients. The 5-year overall survival was 64.7% in the observation group and 88.2% in the adjuvant group (*p* = 0.01). The 5-year disease-free survival was 51.3% in the observation group and 74.0% in the adjuvant group (*p* = 0.011; Figure 1).

### Risk factors for overall survival and disease-free survival

In univariate analysis, age, ECOG, and platinum-based adjuvant chemotherapy were risk factors for overall survival. In multivariate analysis, only platinum-based adjuvant chemotherapy was a risk factor for overall survival [hazard ratio (HR) = 0.428, 95% confidence interval (CI) 0.184-0.998, *p* = 0.049; Table 2]. Platinum-based adjuvant chemotherapy was also a risk factor for disease-free survival when adjusting for age, performance status, and differentiation in multivariate analysis (HR = 0.57, 95% CI 0.263-0.978, *p* = 0.043; Table 3).

**Table 2:** Risk factors for overall survival in stage IB NSCLC

	**Univariate analysis**		**Multivariate analysis (Cox hazard model)**	
	**HR (95% CI)**	***p* value**	**HR (95% CI)**	***p* value**
Age	1.053 (1.015-1.094)	0.006	1.031 (0.995-1.069)	0.092
Sex (female vs. male)	0.571 (0.254-1.284)	0.175	0.689 (0.300-1.584)	0.381
ECOG 1 (vs. 0)	2.343 (1.128-4.864)	0.022	1.717 (0.804-3.668)	0.163
2 (vs. 0)	0	0.976	0	0.977
Pneumonectomy	1.389 (0.421-4.585)	0.590	…	…
Tumor size	0.980 (0.947-1.015)	0.264	…	…
Numbers of dissected lymph nodes	0.987 (0.956-1.018)	0.408	…	…
Adenocarcinoma (vs. non-adenocarcinoma)	0.898 (0.437-1.845)	0.769	…	…
Differentiation (moderate to poor vs. well)	1.317 (0.638-2.944)	0.419	…	…
Lymphovascular invasion	0.494 (0.115-2.110)	0.341	…	…
Pleural invasion (pl2 and pl1 vs. pl0)	1.228 (0.524-2.875)	0.637	…	…
Platinum based chemotherapy (vs. no adjuvant therapy)	0.360 (0.159-0.813)	0.014	0.428 (0.184-0.998)	0.049

**Table 3:** Risk factors for disease-free survival in stage IB NSCLC

	**Univariate analysis**		**Multivariate analysis (Cox hazard model)**	
	**HR (95% CI)**	***p* value**	**HR (95% CI)**	***p* value**
Age	1.032 (1.004-1.062)	0.028	1.022 (0.994-1.051)	0.132
Sex (female vs. male)	0.795 (0.427-1.480)	0.470	…	…
ECOG 1 (vs. 0)	1.679 (0.895-3.149)	0.107	1.552 (0.802-3.002)	0.192
2 (vs. 0)	0.802 (0.107-5.989)	0.829	0.980 (0.113-8.464)	0.985
Pneumonectomy	1.176 (0.421-3.290)	0.757	…	…
Tumor size	0.998 (0.970-1.027)	0.897	…	…
Numbers of dissected lymph nodes	0.984 (0.960-1.010)	0.207	…	…
Adenocarcinoma (vs. non-adenocarcinoma)	1.220 (0.675-2.206)	0.509	…	…
Differentiation (moderate to poor vs. well)	1.675 (0.874-3.211)	0.120	…	…
Lymphovascular invasion	0.827 (0.322-2.124)	0.692	…	…
Pleural invasion (pl2 and pl1 vs. pl0)	0.953 (0.458-1.983)	0.897	…	…
Platinum based chemotherapy (vs. no adjuvant therapy)	0.454 (0.244-0.847)	0.013	0.507 (0.263-0.978)	0.043

### Subset survival analysis for tumor size, performance status, and differentiation

Subset analysis was performed to identify groups of patients who might benefit from platinum-based adjuvant chemotherapy. Platinum-based adjuvant chemotherapy was effective in patients with tumor size greater than or equal to 3.2 cm (median tumor size) (HR = 0.256, 95% CI 0.076-0.854) but was not effective in patients with tumor size less than 3.2 cm (HR = 0.6, 95% CI 0.191-1.887). Adjuvant chemotherapy was also effective in patients with moderate to poor differentiation (HR = 0.222, 95% CI 0.074-0.667) and in patients with ECOG 0 (HR = 0.309, 95% CI 0.108-0.889). There were no survival differences with platinum-based chemotherapy according to the presence of pleural invasion.

**Figure 2:**
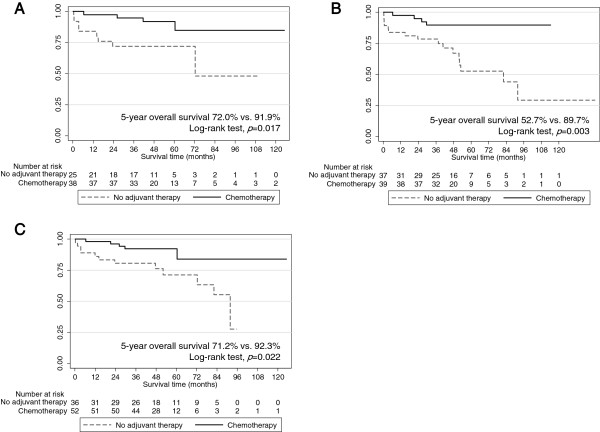
**Survival curves for subset analysis. A**. Overall survival according to adjuvant therapy in patients with tumor size greater than or equal to 3.2 cm (n = 63). **B**. Overall survival according to the adjuvant therapy in patients with moderate to poor differentiation (n = 76). **C**. Overall survival according to the adjuvant therapy in patients with good performance status (n = 88).

In patients with tumor size greater than 3.2 cm (n = 63), the 5-year overall survival was 72.0% in the observation group and 91.9% in the adjuvant group (*p* = 0.017, Figure 2A). In patients with moderate to poor differentiation (n = 76), 5-year overall survival was 52.7% in the observation group and 89.7% in the adjuvant group (*p* = 0.003, Figure 2B). In patients with ECOG 0 (n = 88), 5-year overall survival was 71.2% in the observation group and 92.3% in the adjuvant group (*p* = 0.022, Figure 2C).

## Discussion

This retrospective study showed that platinum-based adjuvant chemotherapy offered better overall and disease-free survival in surgically treated stage IB NSCLC than observation alone. In particular, subset analysis showed that patients with greater tumor size, moderate to poor differentiation, and good performance status (ECOG 0) benefitted from platinum-based adjuvant chemotherapy.

There have been several studies on adjuvant chemotherapy in stage IB NSCLC. Daily administration of the oral agent uracil-tegafur (UFT) for 2 years was proven to be an effective adjuvant treatment for stage I NSCLC. A randomized phase III study of UFT in 978 Japanese patients with stage I adenocarcinoma that was reported in 2003 demonstrated an HR for survival of 0.71 (*p* = 0.04) [12]. The HR for UFT in a meta-analysis of six trials involving a total of more than 2000 patients was 0.74 (*p* = 0.001) [13]. The benefit of UFT was limited to those with tumor size greater than or equal to 2 cm. However, in contrast to the current study, UFT was used as prolonged daily maintenance and, moreover, UFT is not commercially available in North America. With respect to studies of intravenous platinum-based adjuvant chemotherapy, the Cancer and Leukemia Group B (CALGB) trial 9633 was the only randomized adjuvant trial that focused exclusively on patients with stage IB disease. CALGB 9633 trials used paclitaxel with carboplatin and initially planned to involve 500 patients diagnosed with stage IB NSCLC after surgical resection. Accrual to CALGB 9633 was stopped early with 344 patients by the Data and Safety Monitoring Board after a planned interim analysis demonstrated that overall survival had crossed a pre-specified stopping boundary for efficacy, with an HR for overall survival of 0.62 (90% CI 0.44-0.89; *p* < 0.014) [8]. However, the 2006 updated results were no longer statistically significant for overall survival (HR = 0.8, *p* = 0.1). A statistically significant survival advantage was still observed at 2 and 3 years, but statistical significance was lost by 5 years with wide CIs. There are many possible explanations for the final negative results reported with CALGB 9633, including an underpowered study (slow accrual and smaller numbers of patients than expected), a true lack of benefit from adjuvant therapy in patients with stage IB disease, or a true lack of benefit from carboplatin- (as opposed to cisplatin) based adjuvant chemotherapy [14].

Although the efficacy of platinum-based adjuvant chemotherapy in stage IB NSCLC has not been proven, because of the modest overall survival and high recurrence rate for stage IB disease we have recommended and performed platinum-based adjuvant chemotherapy according to the preference of the surgeon and physician if the patient has risk factors for poor survival and is considered able to tolerate treatment. Oral UFT has not been prescribed recently in our institution due to its unavailability. We showed that platinum-based adjuvant chemotherapy offered better overall and disease-free survival in surgically treated stage IB NSCLC. Our results are noteworthy in some aspects compared with previous reports [8,15,16]. First, the latest (7^th^) edition of TNM staging in lung cancer changed the staging criteria for tumor size in T2a from ‘greater than 3.0 cm’ to ‘greater than 3.0 cm and less than 5 cm’ [17]. Previous studies, including CALGB 9633, used the 6^th^ edition of TNM stage in lung cancer and included larger-sized tumors greater than 5 cm, and the range of tumor size in the CALGB 9633 trial was from 0 to 14 cm [8]. In a subset analysis, patients in CALGB 9633 with tumors greater than or equal to 4 cm had an overall survival advantage with an HR of 0.66 (*p* = 0.04) whereas the 74 patients in each arm with tumors less than 4 cm did not show a difference in survival based on treatment with an HR of 1.02 (*p* = 0.51). Despite the exclusion of tumors larger than 5 cm, we detected a benefit of platinum-based chemotherapy in patients with a larger tumor size using criteria based on the 7^th^ edition of TNM stage (more than 3.2 cm). Thus, the benefit of adjuvant chemotherapy was still valid for tumors smaller than 5 cm. Second, we found a benefit of platinum-based chemotherapy according to differentiation and performance status. To our knowledge, previous studies did not perform subset analysis according to these characteristics.

However, this study should be interpreted with caution because of several limitations. First, this is not a randomized prospective study. The patient’s baseline characteristics are different between the two groups: the adjuvant group showed poor pathologic risk factors for recurrence and survival such as poor differentiation and large tumor size whereas the observation group showed poor baseline characteristics for survival such as old age and poor performance status. We considered propensity score matching for comparison but did not perform it due to the small number of subjects after matching. This could be considered a shortcoming of this retrospective study. However, instead of propensity score matching, we adjusted for possible risk factors for overall and disease-free survival such as performance status, age, and sex in multivariate analysis. Despite the poor risk factors for survival and recurrence, the adjuvant group showed better survival than the observation group. Second, as the regimens of chemotherapy were not consistent in the adjuvant group we could not definitely conclude that platinum-based adjuvant chemotherapy had a benefit in stage IB lung cancer. Third, the median follow up periods 49 months are relatively short. However, in lung cancer patients, the recurrences usually develop within 2 or 3 years after operation. We think that median 49 months could reflect the event of recurrence sufficiently given that these early recurrences. Nonetheless, data from this retrospective study warrant a large-scale randomized prospective study of the efficacy of adjuvant chemotherapy in stage IB NSCLC.

What characteristics should be considered in a future randomized prospective trial? First, it must include many more patients than were enrolled in CALGB 9633. Pignon et al. performed meta-analysis of large adjuvant trials for NSCLC conducted since 1995 (excluding CALGB 9633) [18]. Their stage IB subset analysis (1,371 patients) trended toward a benefit of adjuvant treatment (HR, 0.92) but failed to reach statistical significance (95% CI, 0.73-0.95). This meta-analysis implied that the benefit of platinum agent-based adjuvant chemotherapy in stage IB NSCLC, if it exists, is small and would require a prohibitively large trial to be detected. It must be noted that for an HR of 0.8 to be statistically significant, the trial would have required more than 1,000 patients [14]. Since collecting more than 1,000 patients in a single institution is impossible, a multi-center study is required. Second, because of the heterogeneity of stage IB NSCLC, selecting patients at higher risk rather than enrolling the entire stage IB population is the proper strategy [19]. CALGB 9633 reported that patients with tumors greater than or equal to 4 cm showed an overall survival advantage with an HR of 0.66 (*p* = 0.04). Our study demonstrated that in addition to tumor size, other factors such as differentiation and performance status have to be considered. Poor pathologic characteristics that were reported in previous studies should also be considered as an indication for adjuvant chemotherapy in stage IB NSCLC, in particular lymphovascular invasion or pleural invasion. Although several studies showed poor survival in patients with pleural invasion [10,20], no analyses have reported the effect of adjuvant chemotherapy in node-negative T2 tumors according to visceral pleural invasion [21]. Furthermore, applying genomics and proteomics approaches to characterize early lung cancer and determine the risk of recurrence could be considered.

## Conclusion

In conclusion, platinum-based adjuvant chemotherapy for surgically treated stage IB NSCLC might offer better survival than observation alone. In subset analysis, patients with larger tumors, moderate to poor differentiation, and good performance status (ECOG 0) appeared to benefit from platinum-based adjuvant chemotherapy. A large-sized prospective randomized clinical trial is needed to determine its true efficacy.

## Abbreviations

NSCLC: Non-small cell lung cancer; NCCN: National comprehensive cancer network; CT: Chest computed tomography; PET: Positron emission tomography; ECOG: Eastern Cooperative Oncology Group; TNM: Tumor, Node, Metastasis; HR: Hazard ratio; CI: Confidence interval; UFT: Uracil-tegafur; CALGB: Cancer and Leukemia Group B.

## Competing interests

The authors declare that they have no competing interests.

## Authors’ contributions

SYP collected data and write the manuscript. JGL wrote and reviewed the manuscript. JK performed statistical analysis. GEB reviewed medical records and collected data. MKB, CYL, DJK review the manuscript. KYC design the study and review the manuscript. All authors read and approved the final manuscript.
